# Cariogenic Diet Assessment and Analysis Tools in Children and Adolescents: A Systematic Review

**DOI:** 10.3290/j.ohpd.b4997015

**Published:** 2024-02-20

**Authors:** Matina V. Angelopoulou, Kyriaki Seremidi, Vasiliki Benetou, Andreas Agouropoulos, Christos Rahiotis, Sotiria Gizani

**Affiliations:** a Paediatric Dentist and PhD Candidate, Department of Paediatric Dentistry, School of Dentistry, National and Kapodistrian University of Athens, Athens, Greece. Database search, data extraction, calculated risk of bias, drafted the manuscript.; b Paediatric Dentist, Department of Paediatric Dentistry, School of Dentistry, National and Kapodistrian University of Athens, Athens, Greece. Data extraction, calculated risk of bias, edited the manuscript.; c Professor, Department of Hygiene, Epidemiology and Medical Statistics, School of Medicine, National and Kapodistrian University of Athens, Athens, Greece. Wrote part of the manuscript.; d Assistant Professor, Department of Paediatric Dentistry, School of Dentistry, National and Kapodistrian University of Athens, Athens, Greece. Wrote part of the manuscript.; e Associate Professor, Department of Operative Dentistry, School of Dentistry, National and Kapodistrian University of Athens, Athens, Greece. Wrote part of the manuscript.; f Associate Professor and Chair, Department of Paediatric Dentistry, School of Dentistry, National and Kapodistrian University of Athens, Athens, Greece. Conceived the idea for the paper.; All authors read, edited, and approved the final manuscript.

**Keywords:** cariogenic diet, dietary assessment tools, food diary, 24-h recall

## Abstract

**Purpose:**

To collect and evaluate the available evidence on existing tools used in research and clinical practice to assess and analyse the diet of children and adolescents for its cariogenicity.

**Materials and Methods:**

Multiple databases were searched up to October 2022, with no date, publication, or language restrictions, followed by a manual search. Study screening, data extraction, and risk of bias assessment were performed in duplicate. Dietary assessment tools and dental clinical parameters tested were retrieved for qualitative assessment and synthesis.

**Results:**

Of the 2896 papers identified, 9 cohort and 23 cross-sectional studies fulfilled the inclusion criteria. To assess dietary data, 13 studies used a 24-h recall, 11 used a food diary, and 7 used a food frequency questionnaire. For analysis, five studies reported using the Healthy Eating Index, ten used a score based on consumption of sugars, and the remaining analysed cariogenic diet based on the weight and frequency of sugars consumed, or the daily caloric intake from free sugars. Risk of bias assessment suggested that 65.7% of the studies were of moderate and 31.5% of high quality.

**Conclusion:**

Inconsistency exists regarding methods used for the assessment and analysis of dietary cariogenicity. Although every dietary assessment tool has different strengths and limitations, the 24-h recall was the most commonly used method for the assessment of dietary cariogenicity and the most consistent in detecting a positive relationship between sugary diet and carious lesions. A standardised method for cariogenic analysis of dietary data needs to be determined.

Dental caries is the most prevalent chronic disease among children and adolescents^17^ and is the result of an imbalance in the dental biofilm.^32,51^ Frequent consumption of sugars and processed carbohydrates results in an increase of acid-producing microbes in the biofilm found on tooth surfaces. Therefore, individuals with frequent intake of sugars are at a higher risk of developing carious lesions in the long term.^22,32,39^ Finding appropriate ways to collect individualised dietary data and highlighting the effect of daily food intake on a person’s oral health is essential to prevent caries.

Dietary intake can be self-reported either by recording real-time intake using a food diary or by recalling short-term or long-term usual dietary intake using one or repeated 24-h dietary recall/s or a food frequency questionnaire (FFQ).^40^ Τhe 24-h recall assesses food and beverage consumption during the previous day. This tool does not require literacy or numeracy skills as it is typically completed with an interview, is relatively quick, and can be collected even through a telephone call.^29^ However, the 24-h recall relies on short-term memory and is prone to recall bias, and, in some cases, it is challenging to record amounts and portion sizes.^25,29^ The food diary documents food and beverage consumption in real time, usually for three to seven consecutive days. Food diaries do not rely on memory, provide detailed information on every food and beverage consumed, quantitative information on portion sizes, and portray the variation of food selections during the week.^29,44^ On the other, they have a high respondent-burden, require motivation, literacy and numeracy skills as well as adequate training to be written correctly; furthermore, recording of diet may alter habitual dietary behaviours to more socially acceptable and healthy choices. The FFQ records the usual consumption frequency of specific foods and beverages with or without recording their amount.^25^ They can be tailored to target-population characteristics, ethnicity and desired outcome.^29^ Nevertheless, they require some literacy and numeracy skills, usually do not provide information on meal patterns and – if they are not quantitative – they cannot provide information for the calculation of energy and nutrient intake.^25,29^

Each dietary assessment method has its strengths and limitations, and the choice of the most suitable one depends on the purpose of the study and the characteristics of the population involved. However, the food diary in individualised nutrition assessment has been characterised as the gold standard.^25,40^ In dentistry, dietary assessment methods have not been tested for their ability and accuracy in recording data related to oral health. Also, many researchers choose custom-designed questionnaires to evaluate diet. As a result, relevant studies vary considerably, their data assessment methods are not always validated and the findings are not comparable.

The evidence is even more unclear regarding the method used to evaluate collected dietary data for cariogenicity. The first quantified caries-risk dietary analysis traditionally used in clinical practice is based on the calculation of daily production of acids.^41^ This is performed by calculating the total daily frequency of foods and beverages containing sugars and multiplying it by 20, representing the duration in minutes of oral pH drop after each intake. The result is a minute daily score of oral acid production.^41^ Some studies have suggested a score index based on the frequency of consumption of caries-protective, non-cariogenic, or low- and high-cariogenic liquids and solid sugars.^19,35,45,46^ Nevertheless, most studies analyse cariogenic diet based on the daily weight or frequency of sugar consumption, or the daily caloric intake from sugars. Adherence to specific diet quality patterns that provide a measure of the quality of an individual’s overall diet, such as the Healthy Eating Index (HEI), has also been used to detect associations with the risk of caries in childhood.^43,65^ In the HEI, each food category that comprises the index based on the recommendations of the Dietary Guidelines for Americans, is given a score (minimum 0 to maximum 100) based on the fulfillment of minimum-requirement intake, and added sugars and refined grains are included in the estimation.

Overall, evidence is lacking on validated dietary assessment and analysis methods that assess cariogenicity. A systematic review will help evaluate the different methods used to explore dietary data from a dental perspective and define the strength of evidence for each method. Thus, the aim of this systematic review was to collect and evaluate the available evidence on existing tools used to assess and analyse dietary data in relation to caries in children and adolescents.

## Materials and Methods

The Preferred Reporting Items for Systematic reviews and Meta-Analyses (PRISMA) guidelines were followed for the preparation of this review.^27^ The review was conducted according to the Cochrane Handbook,^28^ and the protocol was registered in the PROSPERO international prospective register of systematic reviews (CRD42022302063).

### Study Eligibility Criteria

Randomised controlled clinical trials, prospective and retrospective cohort studies as well as cross-sectional studies were considered eligible for inclusion in this review. In-vitro and animal studies, case reports, editorials and review articles were excluded.

The inclusion criteria for studies were that they:

(1) were performed on healthy children or adolescents aged 0–15 years
(2) assessed dietary habits using traditional or modified/computer-based 24-h recall, food diary/records or a food frequency questionnaire (measuring total meal frequency or frequency of snacks)
(3) analysed diet using a quantitative method
(4) recorded carious lesions using specific criteria (e.g., dmft/DMFT, caries presence or absence).

The exclusion criteria for studies were that they:

1. were performed on participants aged >15 years old, under orthodontic treatment, with eating disorders or medically compromised
2. reported results only for specific food categories (e.g., sugar-sweetened beverages)
3. used qualitative analysis of dietary data or caries
4. were unrelated to diet and oral health
5. did not assess the patients’ dietary habits (e.g., masticatory behaviour).

The population (P) of the study was defined as the children, the intervention (I) was the different diet assessment tools compared (C) to each other, in assessing the outcome (O) of a diet’s cariogenicity.

### Search Strategy

Detailed search strategies were developed and revised for the following seven databases, considering the differences in controlled vocabulary and syntax rules: Medline/PubMed, Cochrane Library, Google Scholar, Embase, Web of Science, Dentistry. The databases were searched up to October 2022. Unpublished literature was searched on grey literature, defined as theses, dissertations, and product reports, and through ClinicalTrials.com, Open Grey, and ISRCTN.

For the search, the following MeSH terms were used: “diet*”, “nutr*”, “food”, “feeding”, “eat”, “sugar”, “taste”, “dent*”, “oral”, “caries”, “decay”, “cavities”, “tooth”, “children”, “infant”, “teenager”, and “adolescent”. A search query for the Pubmed database is presented in Appendix I. Initially, the articles were screened based on their title and abstract using Rayyan software (Hyderabad, India). All articles identified in the search were assessed for relevance to the topic. Irrelevant studies and duplicate studies were excluded. Studies that did not provide enough information for assessment through the title and abstract were placed in the “maybe” category and were assessed based on their full text. The reference lists of the selected articles were also hand-searched for eligible studies not previously identified. No restrictions regarding the date or language of publication were applied in any of the searches.

### Data Collection and Analysis

#### Selection of studies

Study selection was performed independently and in duplicate by two authors (M.A., K.S.) and consisted of title and abstract reading, followed by full-text reading of potentially eligible papers. Disagreements between the authors were resolved by discussion and consultation with a third author (S.G.).

#### Data extraction

Two authors (M.A., K.S.) performed the data extraction independently. The data collected included: (1) publication characteristics (title, authors, date, and country), (2) sample size and age of the participants, (3) study design, (4) oral health variable used, (5) dietary data collection and analysis method, (6) outcome measures and (7) quantitative and qualitative results. The mean and standard deviation (SD) of the dmft/dmfs and/or DMFT/DMFS (Decayed, Missing, Filled Teeth/Surface) index were recorded when available, whereas in the studies that reported caries as present or absent, and odds ratio (OR) or relative risk/risk ratio (RR) of developing carious lesions, the respective value was recorded.

#### Data synthesis

The outcomes were measured by comparing: 1) the mean difference of the dmft/dmfs or DMFT/DMFS index between low- and high-sugar diet groups based on the method used to predict caries risk (sugar score/index, weight of sugars per day, frequency of sugars per day, or daily % of energy from sugars); 2) the presence or absence of carious lesions between low- and high-sugar diet groups; and 3) the risk/odds for caries as consumption of sugars increased.

#### Unit of analysis

In all studies, the unit of analysis was the participant. Some cohort studies presented data from repeated observations on the same participant, resulting in possible unit-of-analysis errors. Section 9.3.4 of the Cochrane Handbook for Systematic Reviews of Interventions was followed in those cases.^28^ Thus, an overall mean was computed for each individual participant at all time points.

#### Missing data

In cases of unclear or missing data, the study’s corresponding author was contacted for clarification.

#### Assessment of heterogeneity

Clinical, methodological and statistical heterogeneity were assessed by examining the characteristics of the studies, the similarity between the types of participants, and the interventions and outcomes as specified in the inclusion criteria.

#### Assessment of reporting biases

Reporting biases (publication bias, duplicate reports, and language bias) were eliminated by conducting an accurate and sensitive search from various sources with no restrictions.

#### Risk of bias within studies

The literature search did not result in the inclusion of any randomised control trials. Thus, the risk of bias in individual studies was assessed using the Newcastle-Ottawa Quality Assessment Scale for Case-Control, Cohort, and Cross-Sectional Studies.^63^ Two authors (M.A., K.S.) assessed each study independently, in duplicate, for selection, comparability, and outcome/exposure. Cohort studies received a maximum of nine stars and cross-sectional studies a maximum of seven stars. Studies receiving 7–9 stars were rated as “good”, 4–6 stars as “fair,” and 0–3 stars as “poor” quality. The lower the study quality, the higher the risk of bias.

#### Summary measures

The primary outcome was to test dietary tools’ ability in detecting diet’s cariogenicity. The secondary outcomes were measured by comparing the presence of carious lesions, prevalence, or risk of carious lesions with low- and high-sugar diets.

## Results

### Search Results

**Fig 1:**
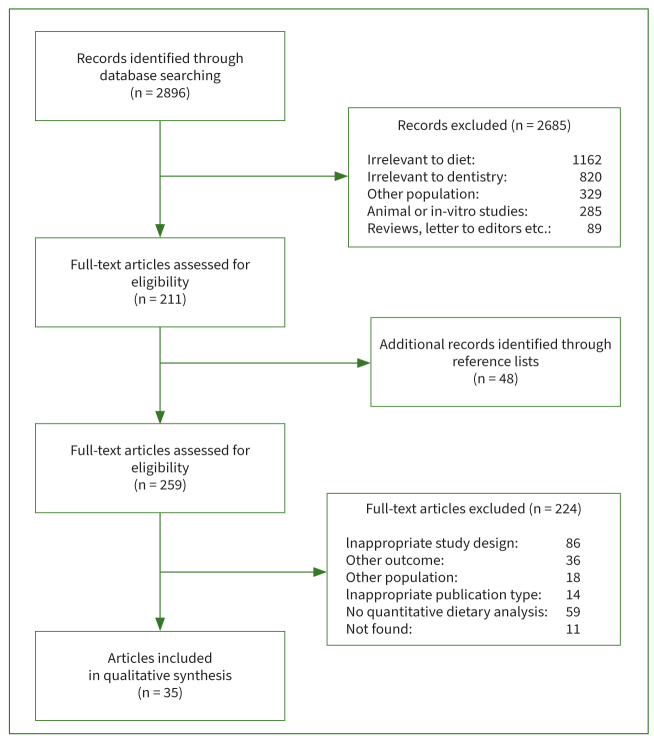
Flow diagram of study selection from all databases.

A total of 2896 records were initially identified through the search of all databases (Fig 1). After the elimination of duplicates, titles and abstracts were reviewed, and 2685 records were excluded. A total of 211 full-text articles were retrieved for assessment and 48 more were added after a hand search. Following full-text evaluation, 224 were excluded with reasoning, and 35 papers were considered eligible. Upon further analysis, it was verified that three groups published two papers with the same cohort, resulting in a total of 32 initial studies which were finally included in the review.

### Study Characteristics

The characteristics of the included papers are summarised in Table 1. Of the 32 studies published between 1959 and 2021, 9 were cohort and 23 cross-sectional. Most studies originated from the USA,^5,10,11,16,33,34,43,45,66^ Brazil,^36,48,53^ Australia,^8,13,31,49^ and the UK^7,12,18,54^ and had been published in the last 20 years (2002–2022).

**Table 1a:** Characteristics (country, sample size and age, caries measurement and comparing factors) of included cross-sectional studies

Author, year of publication	Country	Sample size (age in years)	Dental outcome	Comparing factors
Zita et al, 1959	USA	200 children (5–13)	DMFS	Sucrose consumption
Bagramian et al, 1974	USA	958 children (8)	dmft	Diet
Beighton et al, 1996	UK	328 children (12)	DMFT	Bacteria and diet
Petti et al, 1997	Italy	439 children (6–11)	dmft/DMFT	Sucrose and milk consumption
Nobre DosSantos et al, 2002	Brazil	60 children (1–4)	Caries (yes/no)	Dental plaque composition and daily sugar intake
Cameron et al, 2006	UK	165 children (3–11)	Caries (Hi/Low)	Diet, bowel habit, social class and BMI
Kiwanuka et al, 2006	Uganda	614 children (10–14)	Caries (yes/no)	Validity and reliability of food frequency questionnaire
Amin et al, 2008	Saudi Arabia	1115 children (10–14)	Caries (yes/no)	Dental knowledge, diet and oral hygiene
Nunn et al, 2009	USA	3912 children (2–5)	Caries (dmft>4)	Healthy Eating Index
Palmer et al, 2010	USA	111 children (2–6)	Caries (yes/no)	Bacteria and diet
Parisotto et al, 2010	Brazil	169 children (3–4)	Caries (yes/no)	Dental plaque, socioeconomic level and daily sugar intake
Sankeshwari et al, 2013	India	1250 children (3–5)	Caries (yes/no)	Socioeconomic level and diet
Evans et al, 2013	USA	808 children (2–6)	Caries (yes/no)	Diet
Gupta et al, 2014	India	100 children (12)	dmft/DMFT	Association of BMI, daily sugar intake and oral hygiene
AbdelAziz et al, 2015	Egypt	60 children (2–6)	Caries (dmft>4)	Healthy eating, juice consumption and bacteria
Zaki et al, 2015	Egypt	60 children (2–6)	Caries (dmft>4)	Healthy Eating Index
Palacios et al, 2016	Puerto Rico	1587 children (12)	Caries (yes/no)	Type, amount, and pattern of carbs
Goodwin et al, 2017	UK	128 children (11–12)	Caries (yes/no)	Sugar consumption before bed
Inan–Eroglu et al, 2017	Turkey	395 children (3–6)	dmft	Diet quality
Morikava et al, 2018	Brazil	427 children (5)	Caries (yes/no)	Diet
Pieper et al, 2019	Germany	1019 (9)/ 925 (12)	dmft/DMFT	Sugar Index
Priyasarshini et al, 2019	India	100 children (3–6)	Caries (dmft>4)	Diet
Lee et al, 2020	Malaysia	396 children (3–6)	Caries (yes/no)	Nutritional status, sugars and second–hand smoke
Yang et al, 2021	China	1517 children (12–14)	Caries (yes/no)	Free–sugar intake

**Table 1b:** Characteristics (country, sample size and age, caries measurement and comparing factors) of included cohort studies

Author, year of publication	Country	Sample size (age in years)	Dental outcome	Comparing factors
Rugg-Gunn et al, 1984	UK	405 children (11 to 14)	DMFS	Diet
Burt et al, 1988	USA	499 children (11 to 15)	DMFS	Sucrose intake
Burt et al, 1994	USA	499 children (11 to 15)	DMFS	Sugar intake
Rodrigues et al, 2001	Brazil	510 children (3 to 4)	Dmfs	Sugar intake
Campain et al, 2003	Australia	645 children (12 to 14)	DMFS	Sugar-starch combination
Ruottinen et al, 2004	Finland	66 children (0 to 10)	dmft/DMFT	Sucrose intake
Marshall et al, 2005	USA	634 children (1 to 5)	Caries history (yes/no)	Meal, snack and eating occasions
Marshall et al, 2007	USA	634 children (1 to 5)	Caries history (yes/no)	Sugar intake
Manohar et al, 2009	Australia	738 children (0 to 3)	Caries (yes/no)	Dietary trajectories and obesity
Peres et al, 2016	Australia	302 children (6 to 12)	DMFT	Sucrose intake
Bell et al, 2019	Australia	1170 children (1 to 3)	Caries (yes/no)	Diet quality and risk of obesity

dmft: decayed, missing, filled primary teeth; DMFT: decayed, missing, filled permanent teeth; dmfs: decayed, missing, filled surfaces on primary teeth; DMFS: decayed, missing, filled surfaces on permanent teeth.

The sample size in the included studies ranged from 60 to 3912 subjects, resulting in a total number of 21,752 participants. One-third of the studies (31.4%) had a sample size of up to 200 children,^1,12,18,19,42,45,48,52,55,65,66^ 42.9% had 300 to 700 children,^7,10,11,13,20,23,31,33,34,36,49,53,54^ and 25.7% had a sample of 800 to 4000 children.^3,5,8,16,43,46,47,56,64^

The age range varied from 6 months to 14 years and the mean age was 7.2 ± 4.0 years, with the majority of the studies (46.9%) having sample populations of preschool-age children.

The majority of the studies evaluated the association between diet and caries status, while five studies also evaluated the association of diet with bacterial or dental plaque composition.^1,7,42,45,48^ Five studies assessed the association of diet with caries in combination with other dental and non-dental parameters, such as oral hygiene, socioeconomic status, body mass index, and second-hand smoke,^3,12,19,26,56^ and three studies assessed the association of diet with caries using validity of dietary assessment tools for oral health purposes.^23,43,65^

Thirteen studies compared dietary habits between caries and caries-free children,^8,16,18,23,26,31,33,34,36,42,45,47,48^ five studies assessed diet quality between children with high (dmft/DMFT > 4) and low caries prevalence (dmft/DMFT<4),^1,12,43,52,65^ and the remaining fourteen assessed caries experience by calculating dmft/DMFT index.^5,7,10,11,19,42,46,49,50,52-55,66^

### Risk of Bias within Studies

**Fig 2:**
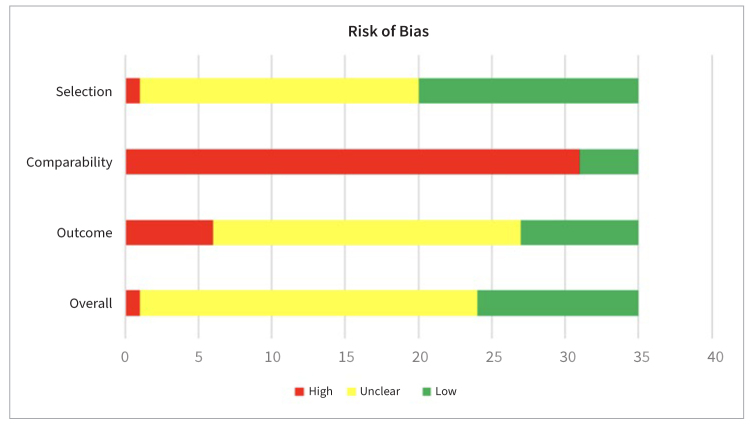
Risk of bias within studies.

Overall, 11 papers were assessed as being at low risk of bias, 23 at moderate risk, and only one at high risk (Table 2, Fig 2). Most studies rated well on the “selection” criterion, and the main reason for the unclear risk was non-reported response rates. In regard to “comparability,” many of the studies did not report control of confounding factors due to the multifactorial nature of caries. They were therefore rated as of unclear risk. Finally, regarding the “outcome” assessment, information on the statistical method used, blindness of the examiner, drop-out rate, and record linkage was missing in six studies which were therefore rated as being at high risk of bias.

**Table 2:** Risk of bias of individual studies assessed by the Newcastle-Ottawa Scale Score

Author, year of publication		Study Design		Newcastle-Ottawa Scale Score	
		Selection	Comparability	Outcome	Total
Zita et al, 1959	Cross-sectional	**	*	**	5/7
Bagramian et al, 1974	Cross-sectional	**	*	*	4/7
Rugg-Gunn et al, 1984	Cohort	****	*	**	7/9
Burt et al, 1988	Cohort	***	*	***	7/9
Burt et al, 1994	Cohort	****	*	***	8/9
Beighton et al, 1996	Cross-sectional	**	**	*	5/7
Petti et al, 1997	Cross-sectional	**	*	**	5/7
Rodrigues et al, 2001	Cohort	****	*	**	7/9
Nobre DosSantos et al, 2002	Cross-sectional	**	*	**	5/7
Campain et al, 2003	Cohort	****	*	***	8/9
Ruottinen et al, 2004	Cohort	****	*	**	7/9
Marshall et al, 2005	Cohort	***	*	***	7/9
Cameron et al, 2006	Cross-sectional	**	*	*	4/7
Kiwanuka et al, 2006	Cross-sectional	**	*	**	5/7
Marshall et al, 2007	Cohort	***	*	***	7/9
Amin et al, 2008	Cross-sectional	**	*	**	5/7
Nunn et al, 2009	Cross-sectional	**	*	**	5/7
Manohar et al, 2009	Cohort	***	**	***	8/9
Palmer et al, 2010	Cross-sectional	**	*	**	5/7
Parissoto et al, 2010	Cross-sectional	**	*	**	5/7
Sankeshwari et al, 2013	Cross-sectional	***	*	**	6/7
Evans et al, 2013	Cross-sectional	**	**	**	6/7
Gupta et al, 2014	Cross-sectional	**	*	**	5/7
Zaki et al, 2014	Cross-sectional	***	*	**	6/7
Abdel-Aziz et al, 2015	Cross-sectional	**	*	**	5/7
Palacios et al, 2016	Cross-sectional	**	*	**	5/7
Peres et al, 2016	Cohort	****	*	**	7/9
Goodwin et al, 2017	Cross-sectional	*	*	*	3/7
Inan-Eroglu et al, 2017	Cross-sectional	**	*	**	5/7
Pieper et al, 2018	Cross-sectional	***	*	**	6/7
Morikava et al, 2018	Cross-sectional	**	*	*	4/7
Priyadarshini et al, 2019	Cross-sectional	**	*	**	5/7
Bell et al, 2019	Cohort	***	**	***	8/9
Lee et al, 2020	Cross-sectional	**	*	*	4/7
Yang et al, 2021	Cross-sectional	***	*	**	6/7

### Analysis of Outcomes

#### Primary outcome

Thirteen papers used 24-h recall, 11 the food diary, and 7 a FFQ (Table 3a). Additionally, three papers used the 24-h recall combined with a FFQ, and one used 24-h recall combined with a food diary. It was noted that the oldest food-diary study used a 7-day diary record,^66^ whereas studies from the 90s onwards considered the 3-day record sufficient. Of these studies, 25.7% detected an association between dietary intake and carious lesions using 24-h recall, 14.3% using the food diary and 11.4% using a FFQ. The percentage of studies finding no association or weak association using 24-h recall was 5.7% compared to 8.6%-11.4% using the food diary or a FFQ. Regarding the FFQs, it should be noted that different questionnaires were used in the included studies, which may have affected the results depending on the applicability of the FFQ to the specific study population, the time frame covered and the items included in the instrument.

**Table 3a:** Dietary assessment method used in each included study

Author, year of publication	Dietary assessment method
Zita et al, 1959	7 days food diary
Rugg-Gunn et al, 1984	3 days food diary
Beighton et al, 1996	3 days food diary
Nobre DosSantos et al, 2002	3 days food diary
Campain et al, 2003	4 days food diary
Ruottinen et al, 2004	3 days food diary
Marshall et al, 2005	3 days food diary
Marshall et al, 2007	3 days food diary
Parisotto et al, 2010	3 days food diary
Sankeshwari et al, 2013	3 days food diary
Lee et al, 2020	3 days food diary
Cameron et al, 2006	10 items FFQ
Kiwanuka et al, 2006	8 items FFQ
Amin et al, 2008	6 items FFQ
Manohar et al, 2009	32 items FFQ
Peres et al, 2016	6 items FFQ
Pieper et al, 2019	6 items FFQ
Yang et al, 2021	12 items FFQ
Bagramian et al, 1974	24-h recall
Burt et al, 1994	24-h recall
Petti et al, 1997	24-h recall
Rodrigues et al, 2001	24-h recall
Nunn et al, 2009	24-h recall
Palmer et al, 2010	24-h recall
Gupta et al, 2014	24-h recall
Zaki et al, 2015	24-h recall
AbdelAziz et al, 2015	24-h recall
Palacios et al, 2016	24-h recall
Goodwin et al, 2017	24-h recall
Inan-Eroglu et al, 2017	24-h recall
Priyasarshini et al, 2019	24-h recall
Bell et al, 2019	24-h recall, 2 days Food Diary
Burt et al, 1988	24-h recall, FFQ
Evans et al, 2013	24-h recall, FFQ
Morikava et al, 2018	24-h recall, FFQ

*FFQ: Food Frequency Questionnaire.

Dietary analysis methods used in assessing caries risk varied greatly. Ten papers used a score based on the FFQ or a “sweet score”,^49^ “sweet index”^19,56^ etc (Table 3b). Ten papers calculated the dietary impact based on the weight of sugars consumed in a day, six based on the frequency of sugar consumption in a day, and three based on the daily “energy” and total calories count from sugar per day. Six papers used an a-priori diet quality index such as “HEI” or the Dietary Guideline Index, while three used multiple methods to analyse dietary data in relation to cariogenicity. Of the studies that analysed diet using the frequency of sugar consumption and those using the HEI, 14.3% and 11.4%, respectively, found an association between dietary intake and caries. The percentage of studies finding no or a weak association between diet and caries using these methods of analysis was 2.9%.

**Table 3b:** Method used to predict risk of caries in each study and relevant outcome measures

Author, year of publication	Diet analysis method	Outcome measures
Burt et al, 1988	Sugar intake in calories/energy	Odds of caries with sugars % of energy
Burt et al, 1994	Sugar intake in calories/energy	Total and between-meal sugars % of calories, eating frequency, and number of sugary snacks with DMFS
Goodwin et al, 2017	Sugar intake in calories/energy	Odds of caries with sugar consumption before bed
Nunn et al, 2009	Healthy eating index	HEI association with early childhood caries
Zaki et al, 2015	Healthy eating index	HEI association with early childhood caries
AbdelAziz et al, 2015	Healthy eating index	HEI association with early childhood caries
Inan-Eroglu et al, 2017	Healthy eating index, KIDMED	Association of diet quality with dmft
Priyasarshini et al, 2019	Healthy eating index	HEI association with early childhood caries
Bell et al, 2019	Dietary Guideline Index	Odds of caries with quality of diet
Petti et al, 1997	Frequency of sugar-based meals	Association of sucrose frequency with dmft/DMFT
Nobre DosSantos et al, 2002	Frequency of sugar-based meals	Association of daily frequency of sugary meals with caries presence
Rodrigues et al, 2001	Frequency of sugar-based meals	Odds of caries with sugary frequency consumption
Manohar et al, 2009	Frequency of sugar-based meals	Rate ratio of caries by sugary foods consumption
Parisotto et al, 2010	Frequency of sugar-based meals	Odds of early carious lesions with increased number of sugary exposures
Lee et al, 2020	Frequency of sugar-based meals	Risk for caries by the daily frequency of sugary exposure
Zita et al, 1959	Weight of sugars between meals	Association of between-meal amount of sugar with DMFS
Rugg-Gunn et al, 1984	Weight of total sugar intake g/d	Association between weight of daily sugar intake with DMFS
Beighton et al, 1996	Weight of total sugars g/d and eating frequency	Daily intake of sugars in grams and sugar eating daily events with DMFT
Campain et al, 2003	Weight of total sugars and starch	Risk for caries by the weight of sugars and starches consumed
Ruottinen et al, 2004	Weight of total sugar intake g/d	Effect of weight of daily sugar consumption in dmft/DMFT in the longterm
Marshall et al, 2005	Weight of total starch and sugar intake g/d	Association of daily intake of sugars in grams with caries history
Marshall et al, 2007	Weight of total sugar intake g/d	Odds of caries with sugar and starch intake in the longterm
Evans et al, 2013	Weight of added sugars g/d and eating frequency	Odds of caries with added sugars g/d and number of eating occasions
Palacios et al, 2016	Weight of total sugar intake g/d	Odds of caries with total sugars g/d
Yang et al, 2021	Weight of free-sugar intake g/d	Odds of caries with daily intake of sugars in grams
Bagramian et al, 1974	Dietary scores	Association of sugar-consumption frequency, pH daily drops, food retention and amount of sugar with dmft
Cameron et al, 2006	Score on food frequency questionnaire	Association of sugar consumption frequency with caries presence
Kiwanuka et al, 2006	Score on food frequency questionnaire	Association of frequency of sugar consumption with caries presence
Amin et al, 2008	Score on food frequency questionnaire	Odds of caries with frequency of sugar consumption
Palmer et al, 2010	Food cariogenicity score	Food consumption frequency and food cariogenicity with caries presence
Sankeshwari et al, 2013	Sweet score	Odds of caries with daily frequency of sugar intake
Gupta et al, 2014	Sweet score	Odds of caries with daily frequency of sugar intake
Peres et al, 2016	Sweet Index	Effect of sugar consumption frequency in DMFT in the long term
Morikava et al, 2018	Score on food frequency questionnaire	Prevalence of caries with cariogenic food consumption
Pieper et al, 2019	Sugar Index	Association of sugar index with dmft/DMFT

In contrast, half of the studies using a score to analyse diet found an association, and the other half found no or a weak association between diet and caries. Most studies using the weight of sugars to analyse dietary data found no or a weak association between diet and dental caries; only 8.6% found a positive correlation between sugar and caries. No differences were detected between studies using “energy” as a method of analysis.

#### Secondary outcome

An association between caries and a high sugar-diet was found in 18 of the included studies (Tables 4 to 6). In contrast, 9 found a statistically significant association with some dietary variables, such as the total daily and between-meals sugar consumption, number of sweets eaten, the weight of added sugars, etc (Tables 4 to 6). Another 9 studies found no association between caries and any of the dietary variables.

**Table 4:** Caries experience using the mean difference of dmft-s/DMFT-S index in children with high- versus low-sugar diet, statistical significance of the difference and conclusions of these studies

Author, year of publication	Study type	Caries index	Low-sugar diet	High-sugar diet	p	Conclusions
Bagramian et al, 1974	Cross-sectional	dmft	2.91	3.61	0.012	Low association between sucrose consumption and caries both at mealtime and between meals
Ruottinen et al, 2004	Cohort	dmft	1.1	2.7	0.177	Higher sucrose intake increases the caries risk
Inan-Eroglu et al, 2017	Cross-sectional	dmft	4.43	5.39	0.450	Healthy eating pattern and high diet quality associated with lower caries prevalence
Pieper et al, 2019	Cross-sectional	dmft	1.51	2.78	<0.001	Sugar Index associated with caries in primary teeth
Ruottinen et al, 2004	Cohort	DMFT	0.5	1.4	0.010	Higher sucrose intake increases the caries risk
Peres et al, 2016	Cohort	DMFT	0.92	1.92	<0.050	The higher the sugar consumption during the life course, the higher the dental caries increment
Pieper et al, 2019	Cross-sectional	DMFT	1.54	1.99	0.054	Sugar Index associated with severe caries in permanent teeth
Petti et al, 1997	Cross-sectional	dmft+DMFT	1.36	3.24	<0.001	Low sucrose-consumption frequency associated with better dental health
Zita et al, 1959	Cross-sectional	DMFS	5.82	7.79	>0.010	No correlation between total sugars consumed and caries
Rugg-Gunn et al, 1984	Cohort	DMFS	3.19	4.97	>0.050	More sugars consumed daily correlated to higher caries; frequency not correlated
Burt et al, 1988	Cohort	DMFS	2.4	3.05	0.150	Frequency of meals and sugary snacks not related with caries Total daily and between-meal sugar consumption associated only with approximal caries
Beighton et al, 1996	Cross-sectional	DMFS	5.9	6.4	<0.050	Total daily starch intake only (not sugar) and total sugar eating events associated with caries
Pieper et al, 2019	Cross-sectional	DMFS	1.75	2.14	0.069	Sugar Index associated with severe caries in permanent teeth

**Table 5:** Dietary score between children with and without caries, statistical significance of the difference and conclusions of these studies

Author, year of publication	Type of study	No caries	High caries	p	Conclusions
Nobre DosSantos et al, 2002	Cross-sectional	2.88	5.32	<0.05	Number of sugar events daily was associated with ECC and affects the composition of dental plaque
Cameron et al, 2006	Cross-sectional	32	30.9	0.21	No association between sugar-consumption frequency and caries
Kiwanuka et al, 2006	Cross-sectional	5.8	5.7	>0.05	No association between sugar-consumption frequency and caries
Marshall et al, 2007	Cohort	95	96	0.785	Sugar consumption alone insufficient to detect dietary impact on caries
Nunn et al, 2009	Cross-sectional	70.5	66.7	<0.001	Healthy eating associated with lower caries risk
Palmer et al, 2010	Cross-sectional	2	2.31	<0.0005	Food frequency and cariogenicity were associated with ECC
Zaki et al, 2015	Cross-sectional	77.6	63.7	<0.0001	Healthy diet has a protective effect against caries
Priyasarshini et al, 2019	Cross-sectional	80.4	44.5	0.000	Healthy diet has a protective effect against caries

**Table 6:** Risk of developing caries (measured by Odds Ratio-OR/Relative Risk-RR) with the different dietary factors, statistical significance of the difference (p-value) and conclusions of these studies

Author, year of publication	OR/RR	p	Conclusions
Burt et al, 1994	1.22	<0.05	High sugar consumption was associated with increased caries on proximal surfaces, caution should be taken with fat substitution of sugar in meals
Rodrigues et al, 2001	6.29	<0.001	High frequency of sugar consumption increases caries risk
Campain et al, 2003	1.23 (RR)	<0.05	Foods that contain relatively little sugar but are high in starch, e.g., bread, muffins, crackers, cereals, and pastries, seem to increase caries risk
Amin et al, 2008	4.7	<0.001	Frequent exposure to cariogenic food (candy, cake, juice, chocolate, sodas etc) associated with higher caries prevalence
Marshall et al, 2005	2.26	<0.05	Higher frequency of eating occasions and higher exposure to sugars within snacks increase caries risk
Manohar et al, 2009	0.9 (IRR)	0.747	No association between high-sugar diet and caries, but statistically significant association with breastfeeding longer than 12 months
Parisotto et al, 2010	5.45	<0.05	Sugars increase the risk for caries
Evans et al, 2013	1.00	<0.05	Sugar sweetened beverage (SSB) intake statistically significantly increases the risk for severe early childhood caries
Sankeshwari et al, 2013	1.51	0.047	Increased sugar-consumption frequency, sucrose exposure between meals and a higher frequency of eating occasions were associated with caries
Gupta et al, 2014	1.214	0.578	No statistically significant effect of daily sugar-intake frequency with prevalence of caries
Palacios et al, 2016	1.88	<0.05	Sugar consumption increases the risk of caries. especially sucrose, fructose and inositol that are often contained in SSBs and fruit juice
Goodwin et al, 2017	3.6	0.002	No association between free-sugar consumption (%) and caries but statistically significant association with consuming sugars before bed
Morikava et al, 2018	1.07 (PR)	0.002	The more cariogenic foods (soft drinks, cookies, candy, ice-cream etc.) consumed in a day, the higher the risk of caries
Bell et al, 2019	0.987	0.257	No association between diet quality and obesity with caries
Lee et al, 2020	1.95 (RR)	0.001	High frequency of sugar consumption increases caries risk
Yang et al, 2021	1.446	<0.01	Increased free-sugar intake is a risk factor for dental caries

OR: odds ratio; RR: relative ratio; IRR: indidence rate ration; PR: prevalence ratio.

Specifically, the average dmft value in the low-sugar diet group (score <10th percentile) was 2.49 compared to 3.62 in the high-sugar diet group (score >90th percentile). In the permanent dentition, the average DMFT value was 0.99 for the low-sugar diet group and 1.77 for the high-sugar diet group. Most studies using the DMFS/dmfs index found a statistically significant association between a high-sugar diet and caries experience (Table 4). The corresponding association at the surface level (DMFS, dmfs) was unclear, and the average DMFS values were 3.81 and 4.87 for the low- and high-sugar groups, respectively.

In the studies that used the HEI to measure adherence to a specific healthy dietary pattern, an average score of 76.2 on HEI was reported in the group of children without carious lesions compared to 58.3 in the caries group. The difference in all these studies was statistically significant (p<0.001), indicating that healthy eating was strongly related to fewer caries (Table 5). The remaining studies that used a dietary score to compare children with and without carious lesions were inconclusive.

Finally, most studies (75%) that used OR or RR to evaluate the association of diet on caries found that sugar consumption increased the risk for caries (Table 6). More specifically OR, varied between 0.73 and 6.29.

### Quantitative Analysis

To test the ability of dietary tools in providing evidence for the causal association of diet with dental caries, longitudinal studies such as cohort studies are required. Nine of the present systematic reviews in this study had a longitudinal cohort design. However, none used the same dietary analysis method; thus, their results could not be combined and compared in a meta-analysis.

## Discussion

This systematic review assessed and summarised the available literature on the various dietary tools used to collect and evaluate data on dietary cariogenicity in children and adolescents. The findings suggest that 24-h recall is the most frequently used dietary assessment method for collecting dietary data for caries risk assessment. Also, dietary analysis of cariogenic diet varied considerably between studies; there is no standardised method used in dentistry. The “sweet score”, the “cariogenicity score”, or the traditional calculation of “oral pH-drop duration” can be used to analyse the dietary data collected.

For this review, we limited the search to children and adolescents, as caries is strongly associated with diet and is the most common oral disease in this age group.^22^ Also, studies reporting the association of consuming specific food groups or categories (e.g., sugar-sweetened beverages, type of infant feeding/breastfeeding) were excluded from the review.

Regarding the caries indices used, only fourteen of the studies used the dmft/DMFT index to assess caries experience.^5,7,10,11,13,19,20,46,49,50,53-55,66^ Since the primary outcome of the study was to collect the available evidence on dietary assessment tools, it was decided to also include the studies that recorded caries based on presence/absence and high/low number of carious lesions, to increase the number of the included studies that may have presented different dietary assessment tools.

Most of the studies included in this review were published in the last 20 years. However, many dental studies on diet were published before that, such as the Vipeholm study in 1959, that established the importance of diet, sugar texture, and frequency of sugar consumption on caries formation.^24^ Conducting clinical studies with a randomised design, e.g., the Vipeholm study, would be unethical; therefore, only observational studies were available and were included in this review.

Nine of the studies in this review had a longitudinal design, which is required to detect the effects of diet on caries. Most of the included cohort studies with long-term evaluation consistently found an association between sugar intake and caries, regardless of the dietary analysis method used. The results of this systematic review stress the need for longitudinal studies using standardised dietary evaluation and analysis tools to obtain more accurate and comparable findings.

The sample size of the population included in all these studies ranged from 60 to 3912 participants, due to the different study design, and the age of the participants ranged from 6 months to 14 years. Regarding age, the sample population of most studies (48.6%) consisted of preschool children; this is probably because the diet of preschool children is usually controlled by the parent/caregiver, so that more accurate data can be easily collected.^59^

Many of the included studies tested other factors in combination with diet and caries, such as oral bacteria, oral hygiene, socioeconomic status, etc.^1,3,7,18,19,26,42,45,48,56^ Caries is a multifactorial disease, and diet is only one of the contributing factors.^9^ Many studies chose to include other factors, in combination with diet, to examine and control for the multifactorial nature of caries. All the studies evaluating diet and microbes in relation to caries have consistently found a positive association.^1,7,31,42,45,48^ In contrast, studies including other risk factors – e.g., socioeconomic status, oral hygiene, or body mass index – that are considered secondary to diet and caries did not always detect correlations/associations with oral health.^12,19^ Children of low-income families and minority populations are at greater risk in developing carious lesions; this association was also found in the studies included in this review.^16,53^ Also, low socioeconomic status is associated with poor diet quality as well as high sugar intake in the need to cover energy requirements with low-cost food items.^14^ The role of fluoride, calcium and phosphate as modulating factors in the caries process needs to be taken into account. Thus, community programs such as water fluoridation, or personal measures of oral hygiene and the use of topical delivery vehicles (e.g., toothpaste/rinses/cremes) can contribute to the complexity of the relationship between diet and caries.

Regarding the dietary assessment tools, as mentioned above, each method has its unique features as well as strengths and limitations.^6,25,40,58^ The food diary or food record is an open-ended method that requires recording of all foods and beverages consumed on a particular day. Traditionally, this was collected for seven days; but as found in this review and as reported in the past, shorter periods (e.g., 3 days) are adequate.^44^ In addition to weekdays, they include one day from the weekend, which is advisable since the diet on weekends differs from that of weekdays.^4,44,61^ Overall, food diaries are more expensive and time-consuming and thus cannot be used in large-scale epidemiological studies or busy practices.^29,44^ In children, the challenge is greater, as the parent usually makes entries in the diary, due to the skills required for its completion. Some data might be missing if food is consumed outside parental presence.^25^ In dental clinical studies, its application is limited and is mainly used to calculate the amount of sugar consumed. Despite its limitations, a 3-day food diary is an excellent tool, and can be chosen in dental practice in cases where more detail is needed to determine the dietary cause of caries in a particular patient.

The 24-h recall was the most commonly used tool to collect dietary data in oral health studies and usually is the tool of choice in large, national dietary surveys.^15^ Compared to other tools, the present work found that the studies which used it were more likely to detect an association between intake of sugars and caries, which is well documented.^37^ The 24-h recall is also an open-ended method, which provides detailed information on intake and meal patterns over the previous day.^35^ However, when it is completed by children, bias can occur due to children’s difficulties in recollecting their previous day.^25^

The FFQ, depending on the items included, can be quick and practical for use in research. Sometimes it is used in everyday clinical practice to provide the clinician a quick preview of the participant’s dietary habits, and in research, it can provide a dietary score that can be statistically analysed in relation to dental outcomes. It might be easier to use with children, as it does not require recording of the consumption on a specific day but rather a documentation of usual habits about which parents/guardians normally have better knowledge.^25^ However, there is no validated, standardised FFQ to evaluate diet from an oral health perspective.

Methods analysing diet cariogenicity differed greatly between studies. Some used a score, some an a priori dietary pattern such as the HEI, some calculated the quantity of sugars or starch consumed, some the frequency of sugar consumption, and others the daily energy and calories consumed. Caries is a multifactorial disease, and various diet-related factors, such as the number of meals, frequency, quantity, timing, length of exposure, food texture, etc. can contribute to carious lesions formation.^35^ There are no standardised tools in dentistry to evaluate nutrition from an oral health perspective, considering all these contributing factors.^60^ The World Health Organization guidelines suggest a threshold of sugars for caries based on the % of energy from sugar intake.^38^ Furthermore, the HEI, which has also been suggested and has been associated with lower numbers of carious lesions,^43,65^ is an excellent index to assess the overall diet of the individual. However, it is only one of the many diet-quality indices that are used to assess adherence to a healthy dietary pattern and, by definition, it does not evaluate specific dietary factors or sugar intake, which are important for oral health.

With currently available data, it can be suggested that the appropriate tool should be chosen among the validated tools, depending on the target population and the desired aim and outcome.^58^ In clinical practice, the 3-day food diary or 24-h recall can provide all the information necessary to evaluate a participant’s diet. For children and adolescents, the parents should be involved in the process to guarantee higher completion accuracy.^25^ The “sweet score”, cariogenicity score, or the conventional calculation of oral pH-drop duration can be used to analyse the dietary data collected,^19,35,41,45,62^ and suggestions tailored to the participant can be provided.

In research and large-scale epidemiological studies aiming to assess participants’ usual/habitual dietary intake, an FFQ would be easier to collect and statistically analyse, as it provides numerical data.^4,29^ However, it is recommended that oral health researchers should first validate the oral health FFQ to allow compariston of large data volumes.

Regarding the association of diet with the development of carious lesions, most studies found a positive association with diets high in sugar, which is in accordance with previous literature reviews.^37,38,57^ Two studies that did not report an association between carious lesions and diet and another two that found a correlation only for some dietary variables were published before 2000. This difference may be partially attributed to the different caries detection criteria before WHO and ICDAS criteria standardisation.^21^ Especially for preschool children, non-cavitated lesions are considered indicative of high caries risk and should be taken into consideration.^38^ A high HEI was associated with fewer carious lesions, suggesting that adherence to a healthy dietary pattern is a strongly related factor.^43^ Healthy eating patterns are characterised by high consumption of vegetables, seafood, and whole-grain carbohydrates, which are considered foods and nutrients with low cariogenicity. The evidence was inconclusive concerning frequency of sugar consumption alone, which highlights the complex association of diet with caries and shows that evaluating diet to determine caries risk using only one parameter might be inadequate.

Risk of bias results suggest that most of the studies were of moderate quality, which is a limitation of this study. This is mainly due to the fact that most studies had a cross-sectional design, presenting a higher risk of methodological errors than cohorts and randomised control trials.^30^ Also, the quality of reported dietary data is often not good, and combinations or types of sugars which impact food cariogenicity are missing, making the comparison of these data challenging. Finally, due to the marked variability between the studies, a meta-analysis could not be performed, which is also considered a limitation of this review.

However, this is the first attempt to evaluate the available dietary tools for children’s oral health in a systematic review. There were some cohort studies with a long-term evaluation of caries up to ten years later and with a large sample size of more than 1000 children. The results concerning the sugars’ intake effect on incidence of caries were consistent in the majority of the studies despite their variability. Also, we included only studies that used validated quantitative dietary assessment methods, such as the 24-h recall, the FFQ, or the food diary,^25^ and quantitative data for caries.

## Conclusions

The results of this systematic review suggest that large variability exists between the studies regarding the methods used for dietary data collection and analysis from a caries risk perspective. The 24-h recall was the most commonly used method to collect dietary data for cariogenicity assessment in children and the one most likely to detect an association between caries and a sugary diet. The food diary is a valid tool and can be used in dental cases that require more precision and details on dietary intake assessment with the prerequisite that the individual has the appropriate age and literacy level to perform this task. The FFQ can be a valuable tool in dental research, but a standardised questionnaire needs to be established to acquire valid results and be used for comparative purposes. Regarding the use and analysis of dietary data in dentistry, a standardised method for predicting caries risk must be developed, in order to increase the use and utility of dietary information collected in everyday dental practice and to allow comparisons across studies. In addition, many dieticians currently use technology-based tools, either computer software or a mobile app to assess their patients’ diet. Their use in dentistry is limited to a game app and an information app.^2,67^ A tool for recording and analysing diet from an oral perspective would be valuable in order to help patients evaluate and improve their oral health.

## Appendix

**Appendix I:** PubMed Search History-Diet and Caries 20.10.2022

Search number	Filters	Search details	Results
1		“carie”[All Fields] OR “dental caries”[MeSH Terms] OR (“dental”[All Fields] AND “caries”[All Fields]) OR “dental caries”[All Fields] OR “caries”[All Fields] OR (“decay”[All Fields] OR “decayed”[All Fields] OR “decaying”[All Fields] OR “decays”[All Fields]) OR (“cavity s”[All Fields] OR “dental caries”[MeSH Terms] OR (“dental”[All Fields] AND “caries”[All Fields]) OR “dental caries”[All Fields] OR “cavities”[All Fields] OR “cavity”[All Fields])	377,793
2		“caries”[Title/Abstract] OR “decay”[Title/Abstract] OR “cavities”[Title/Abstract]	158,410
3		(“caries”[Title/Abstract] OR “decay”[Title/Abstract] OR “cavities”[Title/Abstract]) AND (“diet”[Title/Abstract] OR “food”[Title/Abstract] OR “eat”[Title/Abstract])	4,319
4		(“caries”[Title/Abstract] OR “decay”[Title/Abstract] OR “cavities”[Title/Abstract]) AND “diet*”[Title/Abstract]	4,418
5	Child: birth-18y	((“caries”[Title/Abstract] OR “decay”[Title/Abstract] OR “cavities”[Title/Abstract]) AND “diet*”[Title/Abstract]) AND (allchild[Filter])	1,569
6		(“caries”[Title/Abstract] OR “decay”[Title/Abstract] OR “cavities”[Title/Abstract]) AND “diet*”[Title/Abstract] AND “child*”[Title/Abstract]	1,412
7		“dental caries”[MeSH Terms] AND (“diet*”[Title/Abstract] OR “eat”[Title/Abstract])	2,896
8		“dental caries”[MeSH Terms] AND (“diet*”[Title/Abstract] OR “eating”[Title/Abstract])	3,175
9		(“caries”[Title/Abstract] OR “decay”[Title/Abstract] OR “cavities”[Title/Abstract]) AND (“diet*”[Title/Abstract] OR “nutri*”[Title/Abstract])	6,445
10		“nutrients”[MeSH Terms] AND “dental caries”[MeSH Terms]	274
